# Socioeconomic Factors Associated with Psychoactive Substance Abuse by Adolescents in Serbia

**DOI:** 10.3389/fphar.2017.00366

**Published:** 2017-06-13

**Authors:** Katarina M. Janicijevic, Sanja S. Kocic, Svetlana R. Radevic, Mirjana R. Jovanovic, Snezana M. Radovanovic

**Affiliations:** ^1^Department of Social Medicine, Faculty of Medical Sciences, University of KragujevacKragujevac, Serbia; ^2^Department of Psychiatry, Faculty of Medical Sciences, University of KragujevacKragujevac, Serbia

**Keywords:** psychoactive substances, adolescent, socioeconomic factors, national health survey, Serbia

## Psychoactive substance abuse in adolescency

Adolescence is a period of transition from childhood to adulthood, characterized by efforts to achieve the objectives related to the expectations of the culture, as well as the requirements of the physical, mental, emotional, and social development. It has its own characteristics in the biological, psychological, and social terms, with a process of identity formation, the development of social, and moral norms of behavior (McCabe et al., 2017). Adolescence is a period of great settings in which experimentation with psychoactive substances is common and can, in some cases, lead to the development of long-term addictive behavior (Jeannin et al., 2013). Psychoactive substances include licit, illicit, and prescribed psychoactive medications. Alcohol and cigarette are among the licit and controlled drugs, while marijuana, cocaine, heroin, lysergic diethylamide (LSD), crack, and ecstasy are illicit drugs (Kassa et al., 2014). The use of psychoactive substances in adolescents is often associated with a socioeconomic factors, such as gender, age, type, race, ethnicity, family, and social structures, socioeconomic status of the family (Gebreslassie et al., 2013).

Significant risk factors for psychoactive substances use among adolescents were poor parental involvement in the child's education, conflictual family relationships, and drug abuse by the parents, friends, and neighbors (Kpozehouen et al., 2015; Pisarska et al., 2016). Also, parental alcoholism, parental divorce before age 18, and parental death before age 18 increased the odds of abuse psychoactive substances (Vaughan et al., 2017).

Socioeconomic environment in which young people were raised as children predicts their behavior in young adulthood. Understanding this relationship, is an important step in identifying persons at risk (Tobler et al., 2000). In order to identify the risk factors and protective factors associated with psychoactive substances abuse among young people, it is very important to measure how socioeconomic factors influence the attitudes and behavior of young toward the use of psychoactive substances (Carter et al., 2010; Patrick et al., 2012).

## The data report methods

### Public data set description—Serbian 2013 National Health Survey

The study of health of population in Serbia conducted in 2013 was the source of used data. This was the third national population health survey conducted by the Ministry of Health of the Republic of Serbia^1^. In the third Survey, a harmonization of research tools (methodology, questionnaires, instructions) with the instruments of the European Health Survey second wave (EHIS wave 2)^2^ was carried out in order to achieve the highest degree of comparability of results with the countries members of the European Union, according to a defined, internationally accepted indicators (ECHI, OMC, WHO, UNGASS, MD).

Fieldwork was conducted in the period from 7 October to 30 December 2013, which respected the legislation relating to the European Health Research—second cycle: the collection of data in the field should take at least 3 months of which at least 1 month should be in the period from September to December, or in the fall. In order to achieve a high level of quality of the collected data, to provide a high response rate of households and in order to protect the representativeness of the sample, the election, and training of interviewers had been organized prior to the commencement of field work, and also guidelines for the monitoring and control of field work were given to them.

The study used the most complete population register that includes a sampling units defined within the target population—Census of Population, Households, and Dwellings in the Republic of Serbia conducted in 2011. In accordance with the recommendations for the implementation of population health research EUROSTAT, the European Health Research—Second Wave—Methodological guide (EHIS wave 2, Methodological manual) the National representative probability sample was used: two-stage stratified sample with a known probability of selection of sample units at every stage sampling. The sample was drawn so as to provide a statistically reliable estimation of a great number of indicators of population health condition at the national level, considering geographic areas/statistical regions of Belgrade, Vojvodina, Sumadija, Western, Southern, and Eastern Serbia, and at the level of urban and other settlements/areas. The mechanisms that have been used to obtain a random sample of households and respondents represent a combination of the two sampling techniques: stratification and multi-stage sampling.

Health Survey of the Serbian population was carried out through interviews, anthropometric measurements, and blood pressure measurements. Three types of questionnaires were used in the survey: Questionnaire for Household—collecting information on all household members, the characteristics of the household, as well as on the characteristics of the household residence. The questionnaire had to be completed in the course of verbal communication between the interviewers and interviewees who represented the main person in the household to answer questions of interest. The questionnaire “face to face” is to be filled in with each member of the household. Self-administered questionnaire which should be filled in by each household member aged 15 and over without the participation of the interviewer.

This type of questionnaire was used because it was estimated that the questions concerning sensitive items of alcohol use, sexual behavior, and so on were not suitable for filling by method “face to face.” In order to complete the questionnaires a method of computer-assisted personal interviewing—CAPI was used as well as the process of interviewing through paper-and-pencil procedures—PAPI for self-administered questionnaire.

Ethical Standards in Health Research were harmonized with the international World Medical Association Declaration of Helsinki. This study was approved by the competent territorial Ethics Committee of the four major regions of Serbia, based in the Republic Institute of Public Health Batut in Belgrade, Institute of Public Health Novi Sad, Kragujevac and Nis.

In order to respect the privacy of the subjects of research and confidentiality of information collected, all necessary steps were taken in accordance with the Law on Personal Data Protection (Off. Gazette of RS No 97/08, 104/09)^3^. Field researchers were required to give a printed document that informed research participants about the Research (Notice of Survey signed by the Minister of Health) and the approval of the Ethics Committee on its implementation, on the rights of patients, and about where and how they can submit complaint/grievance if estimate that their rights have been in any way compromised. Also, interviewers needed to obtain the signed informative consent of each of the participants for accepting to participate in the survey. In research, the collection of data that identify the respondents was avoided to the greatest possible extent (necessary identifiers were removed at the earliest stage of statistical analysis and replaced with code).

### Survey data description

In the Serbian National Health Survey 2013, a total of 6,500 households and 13,756 participants aged 15 and over were interviewed. Out of total of 10,089 households contacted, 6,500 of them agreed to participate in the survey, so that the response rate of households was 64.4%. Out of total of 16,474 registered household members aged 15 and over, 14,623 of them agreed to be interviewed, giving a response rate of 88.9%. Out of this number of people who agreed to be interviewed, 13,756 of them accepted to complete the self-administered questionnaire (response rate 94.1%). For the purposes of this study, we analyzed data on respondents aged 15–24 years (1,722 interviewed respondents).

Of the independent variables, the researchers used demographic characteristics (age, gender, type of settlement, region) and socioeconomic status (education, employment, and well-being index). Participants' age was categorized in to two age groups (15–19 years; 20–24 years). Gender is coded as male and female, place of residence as urban and rural, regions of Belgrade, Vojvodina, Sumadija, Western, Southern, and Eastern Serbia. Variables that reflect the socioeconomic situation are education, which is designated as higher, secondary, and elementary, employment status as employed and unemployed and household. The Wealth Index is based on household assets and housing characteristics, such as the possession of color TV set, cell phone, refrigerator, dish washer, washing machine, PC, AC, car, construction material of floors, roofs and walls, the number of bedrooms per household member, type of drinking water resources, and sanitation facility as well as heating fuel and Internet access. Based on the Wealth Index, households were classified into five groups of equal size—quintiles: (1) the poorest (Q1), (2) poorer (Q2), (3) middle (Q3), (4) richer (Q4), and (5) the richest (Q5). For the purposes of this paper, respondents were classified into three socio-economic categories: poor class, middle class, and rich class. As the dependent variable in this analysis were used: cigarette smoking (daily and occasional), alcohol abuse, and abuse of other psychoactive substances (drugs and illicit drugs).

The data set has been submitted in a public repository Figshare and it is available on: https://figshare.com/s/f55d3b9213f41fe79827. Data has been uploaded as Excel file while questionnaires are in PDF formats. Readers can retrieve and reuse publicly available information by visiting links given above.

## Description of national survey outcomes

A total of 1,722 participants aged 15–24 years (Mean = 19, *SE* = 2.1 years) were included in the study. There were 51% women in the sample. The highest percentage of respondents has completed secondary education (55.9%), while there is the least of those who have high education (4.6%). In relation to the employment status the highest percentage belongs to the group of inactive or unemployment population (87.6%). More than half of the respondents live in urban areas (55.5%). When it comes to well-being index, the largest percentage of respondents belongs to the rich class (42.4%) followed by those who belong to the poor class (38%) and the middle class (19.6%).

The study depicted that in the past 12 months of the study period 72.9% smoked cigarettes (daily and occasional), 56.5% used alcohol, and 24.2% abused drugs. The prevalence of illicit psychoactive substances such as cannabis, ecstasy, LSD, cocaine, crack, heroin was 0.8%.

Binary logistic regression analysis has not shown a statistically significant impact of examined factors on the prevalence of cigarettes smoking. The only factor that is associated with the consumption of cigarettes is self-assessment of health, respondents who evaluate their health as good for 30% less often smokers than in those who evaluate their health as poor (*OR* = 0.700). Results of binary logistic regression showed that alcohol consumption can be determined by age, gender, education, type of settlement, well-being index physical, and psychological violence. The prevalence of alcohol consumption in men is 55.2%, whereas men are 1.8 times more likely to use alcohol than women (*OR* = 1.882). Compared to the younger population (15–19 years) members of the 20–24 age group are more likely to consume alcohol (*OR* = 0.508). Respondents with higher education have 55.3% greater chance of alcohol consumption compared to those with low education (*OR* = 0.477). Young people who live in urban areas are 1.8 times more likely to consume alcohol in relation to those who live in rural areas (*OR* = 1.843). Members of poor class for 33.9% less consume alcohol (*OR* = 0.661) compared with those who belong to the rich class of the population. Young people who are exposed to physical violence (*OR* = 6.702) and the physical (*OR* = 5.026) and mental bully (*OR* = 3.405), significantly more frequent alcohol consumption.

As the most important predictors of use of drugs/ illicit drugs were found to be gender and self-assessed health. Men 88% less likely to use drugs and illicit drugs than women (*OR* = 0.120). Also those who assess their health as good to make less frequently by 56.9% compared to those who assess their health as poor (*OR* = 0.431; Table 1).

**Table 1:** Bivariate logistic regression analysis showing socioeconomic correlates of psychoactive substance abuse by adolescents in Serbia.

**Variables**	**Cigarette smoking**		**Alcohol use**		**Drugs/Illegal drugs abuse**	
	**OR (95% CI)**	***p***	**OR (95% CI)**	***p***	**OR (95% CI)**	***p***
Gender^a^	0.900 (0.594–1.364)	*p* = 0.620	1.882 (1.535–2.306)	*p* < 0.001	0.120 (0.032–0.442)	*p* < 0.001
Age (years)	0.954 (0.621–1.467)	*p* = 0.832	0.508 (0.415–0.623)	*p* < 0.001	1.244 (0.400–3.867)	*p* = 0.707
Education^b^	1.185 (0.806–1.742)	*p* = 0.389	0.477 (0.396–0.574)	*p* < 0.001	0.867 (0.331–2.271)	*p* = 0.771
Emplyment status^c^	1.227 (0.701–2.148)	*p* = 0.473	1.335 (0.979–1.821)	*p* = 0.068	1.954 (0.249–15.320)	*p* = 0.524
Type of settlement^d^	0.940 (0.616–1.433)	*p* = 0.773	1.843 (1.504–2.258)	*p* < 0.001	0.686 (0.208–2.265)	*p* = 0.536
Well-being index^e^	1.222 (0.962–1.552)	*p* = 0.101	0.661 (0.580–0.752)	*p* < 0.001	1.013 (0.544–1.883)	*p* = 0.969
Self-assessed health^f^	0.700 (0.501–0.978)	*p* < 0.05	0.864 (0.734–1.017)	*p* = 0.079	0.431 (0.149–1.248)	*p* < 0.05
Exposure to physical violence (in the family, in school, on the street)	1.964 (0.674–5.772)	*p* = 0.216	6.702 (2.869–15.656)	*p* < 0.001	0.334 (0.069–1.612)	*p* = 0.172
Exposure to psychological violence (in the family, in school, on the street)	0.722 (0.225–2.316)	*p* = 0.584	2.210 (0.990–4.932)	*p* = 0.053	0.447 (0.054–3.693)	*p* = 0.455
The tendency to psychological violence	1.966 (0.814–4.751)	*p* = 0.133	3.405 (2.052–5.651)	*p* < 0.001	0.366 (0.094–1.430)	*p* = 0.148
The tendency to physical violence	0.937 (0.475–1.850)	*p* = 0.851	5.026 (2.839–8.896)	*p* < 0.001	0.280 (0.072–1.087)	*p* = 0.066
Risky sexual behavior	0.873 (0.546–0.873)	*p* = 0.572	1.383 (1.016–1.882)	*p* < 0.05	1.211 (0.322–4.563)	*p* = 0.777

a *Reference values for women*.

b *Reference values for low education*.

c *Reference values for employment*.

d *Reference values for urban*.

e *Reference values for poor class*.

f *Reference values for poor self-perceived health*.

## Comparison with published evidence

Psychoactive substances abuse represents a significant problem of the individual, family, and society, leaving a lot of effects on mental and physical health (Milovanovic et al., 2016), family relationships, work ability, and social activities (Jovanovic and Jakovljevic, 2015). There are also significant costs borne by society due to the direct and indirect consequences of abuse and dependence on certain substances (Jakovljevic et al., 2014). Drug abuse is a global problem, and methods of use and consequences of individual and socio-cultural are specific. The consequences of the abuse of substances may be various: education and unemployment, reduced work productivity, poor health, higher rates of human immunodeficiency-HIV and hepatitis B, C infections (Jakovljevic et al., 2013a), social dysfunction, higher rate of violence, poverty, homelessness, a lower probability of recovery, poor treatment outcomes, and poor quality of life (Jakovljevic et al., 2013b). According to the World Health Organization, alcohol, and tobacco are the most commonly abused substances (World Health Organization, 2013, 2014). Eastern Europe and the Balkans region report the high rates of alcohol abuse (Jovanovic and Jakovljevic, 2011). Alcohol abuse is a health problem that significantly contributes to the global disability (liver-diseases, cardiovascular diseases, traffic accidents, fights, murders, suicides; Jakovljevic et al., 2015a), but also it is an economic problem (absenteeism, unemployment, reduced productivity, long-term treatment) (Jakovljevic et al., 2011). Psychoactive substances present a great challenge of public health issue worldwide particularly regarding to the social vulnerable population of adolescents (Jakovljevic et al., 2015b).

According to the results of the 2013 Serbian National Health Survey, distribution of smoking in adults population was 35.8%, daily using alcohol was 4.7%, while using of sedative, for sleeping and analgetics was 61.4%. Illegal drugs were used by less of 1% adults^1^. Aforementioned dataset showed that there are significant differences in the abuse of psychoactive substances among young people in Serbia, depending on the demographic and socioeconomic characteristics of the respondents. They are consistent with the findings of other studies that show that there is no difference between the sexes for cigarette smoking and experimentation with drugs. It is more connected with young men. Young people of lower age groups and those who attend the school are negatively associated with the abuse of cigarette, alcohol, and illicit drugs (Patrick et al., 2012; Malta et al., 2014).

Other studies have shown that young people with low levels of education are regarded as high risk for consumption psychoactive substances (Quek et al., 2013). Anxiety, low self-esteem, and self-control, as well as the low level of parental control also poses a risk for abuse belt (Roy et al., 2015). Characteristics of mental health, such as loneliness and insomnia are positively associated with the abuse of tobacco, alcohol, and illicit drugs. The lack of a friend is a positive correlation with the abuse of tobacco, and illicit drugs, and the negative with the abuse of alcohol (Malta et al., 2014). Also young people who abuse psychoactive substances, are more likely to have higher levels of psychological stress and decreased levels of self-efficacy to resist peer pressure (Champion et al., 2016). Other studies have in turn shown that the lower the level of education of parents associated with a higher risk of psychoactive substances abuse (Johnston et al., 2011), while the characteristics of the family in the sense that they live with their parents and have a parental control (when parents know what the child is doing in his spare time) negatively associated with such a high-risk behavior (Malta et al., 2014). Heavy episodic drinking are frequent among young people who live in incomplete families (Patrick et al., 2012).

Some studies have shown that there is no significant difference in substance abuse between urban and rural areas, but there is the presence of higher levels of knowledge about the psychoactive substances in urban areas (Martinotti et al., 2015). Other studies show that adolescents who live in urban areas significantly more abuse the psychoactive substances in relation to their peers who live in rural areas (Pawłowska et al., 2014).

Many studies that have investigated the correlation of demographic and socioeconomic variables with the abuse of psychoactive substances have shown that high degree of religiosity, higher parent's education living with one or both parents reduces the chance to abuse, while high the socioeconomic status of the family increases the likelihood of psychoactive substances use (Goodman and Huang, 2002; Hanson and Chen, 2007; Schoenborn and Adams, 2010). Children who come from wealthier families with higher socioeconomic status may be at increased risk for the abuse psychoactive substances which can be explained by the fact that their experience more pressure achievement combined with isolation of parents who have careers more demanding. In addition, parents with higher socioeconomic status in comparison with those in the lower socioeconomic status families can have positions that are tolerant of the substance abuse (Luthar and Goldstein, 2008). The higher income families may be related to the use of psychoactive substances because of increased access to, or to buy the substance and have a social association with others who also have financial resources. On the other hand, a lower revenue may be associated with the abuse of psychoactive substances such mechanism of survival due to increased stress and less access to alternative actions that can be a focal point for preventive strategies (Goodman and Huang, 2002). Despite worldwide concern and education about psychoactive substances, many adolescents have limited awareness of their adverse consequences (Oshodi et al., 2010).

## Conclusive remarks

Preventive activities should be carried out through the development of specific programs to promote healthy lifestyles, strengthening the implementation of existing programs, and the promotion of prevention through various forms of educational activities, including peer education, supporting youth initiatives for the implementation of actions aimed at the affirmation of healthy lifestyles, develop social skills, informing young people, and parents about the risks of consuming psychoactive substances through school programs and workshops in schools, identification, and reduction of risk factors in the school environment.

In perceiving the frequency of using illegal drugs, it should take in mind specific limited researches of health in national population, because the drugs abuse, as social non acceptable behavior endanger sincerity of patients during answering these questions.

## Author contributions

All authors listed, have made substantial, direct and intellectual contribution to the work and approved it for publication. KJ and SMR drafted the manuscript. SRR, SK, and MJ contributed through data analysis and interpretation.

### Conflict of interest statement

The authors declare that the research was conducted in the absence of any commercial or financial relationships that could be construed as a potential conflict of interest.

## Footnotes

^1^Institute of Public Health of Serbia “Dr Milan Jovanović Batut.” Results of the National Health Survey of the Republic of Serbia 2013. Available online at: http://www.batut.org.rs/download/publikacije/2013SerbiaHealthSurvey.pdf

^2^European Health Interview Survey (EHIS wave 2) Methodological manual 2013. Available online at: http://ec.europa.eu/eurostat/documents/3859598/5926729/KS-RA-13-

^3^Law on Personal Data Protection Off. Gazette of RS No 97/08. Available online at: http://www.paragraf.rs/propisi/zakon_o_zastiti_podataka_o_licnosti.html
